# Quantification of nanoparticles' concentration inside polymer films using lock-in thermography†

**DOI:** 10.1039/d3na00091e

**Published:** 2023-04-27

**Authors:** Giulia Mirabello, Lukas Steinmetz, Christoph Geers, Barbara Rothen-Ruthishauser, Mathias Bonmarin, Alke Petri-Fink, Marco Lattuada

**Affiliations:** a Department of Chemistry, University of Fribourg Chemin du Musée 9 1700 Fribourg Switzerland marco.lattuada@unifr.ch; b Adolphe Merkle Institute, University of Fribourg Chemin des Verdier 4 1700 Fribourg Switzerland; c NanoLockin GmbH Route de la Fonderie 2 1700 Fribourg Switzerland; d School of Engineering, Zurich University of Applied Sciences Technikumstrasse 71 8400 Winterthur Switzerland

## Abstract

Thin nanocomposite polymer films embedding various types of nanoparticles have been the target of abundant research to use them as sensors, smart coatings, or artificial skin. Their characterization is challenging and requires novel methods that can provide qualitative as well as quantitative information about their composition and the spatial distribution of nanoparticles. In this work, we show how lock-in thermography (LIT) can be used to quantify the concentration of gold nanoparticles embedded in polyvinyl alcohol (PVA) films. LIT is an emerging and non-destructive technique that measures the thermal signature produced by an absorbing sample illuminated by modulated light with a defined frequency. Films with various concentrations of gold nanoparticles of two different sizes have been prepared by evaporation from homogeneous aqueous PVA gold nanoparticle suspensions. When the thin films were illuminated with monochromatic light at a wavelength close to the plasmonic resonance signature of the nanoparticles, the amplitude of the thermal signature emitted by the nanoparticles was recorded. The measurements have been repeated for multiple modulation frequencies of the incident radiation. We have developed a mathematical method to quantitatively relate the concentration of nanoparticles to the measured amplitude. A discussion about the conditions under which the sample thickness can be determined is provided. Furthermore, our results show how LIT measurements can easily detect the presence of concentration gradients in samples and how the model allows the measured signal to be related to the respective concentrations. This work demonstrates the successful use of LIT as a reliable and non-destructive method to quantify nanoparticle concentrations.

## Abstract 2

We employ lock-in thermography (LIT) to quantify the concentration of gold nanoparticles embedded in polymer films through a mathematical method developed to relate the concentration of nanoparticles to the measured LIT temperature amplitude.

## Graphical abstract

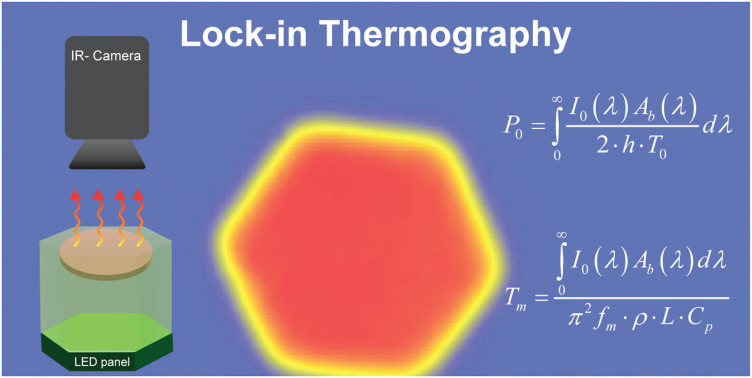

## Introduction

Composite materials have been investigated for decades.^1^ By taking advantage of the different characteristics of their constituents, scientists have developed numerous strategies to prepare composite materials. The advent of nanotechnology has further pushed forward their design, leading to nanocomposites, which are built using nanomaterials.^2^ Exploiting some of the unique properties of engineered nanoparticles (NPs) (such as superparamagnetism,^3^ surface plasmon resonance,^4–7^ quantum dots' optical properties,^8,9^ and the exceptional mechanical resistance offered by carbon nanotubes^10^ and graphene nanoflakes^11,12^), a series of novel materials have been prepared.^3,5,6,8–11,13–17^ This has led to the preparation of a plethora of sensors,^18–20^ functional surfaces,^21–23^ artificial skins^24–26^ and conducting polymer layers,^27–30^ just to mention a few. However, many NPs can only be synthesized through complex procedures, which makes them expensive.^31^ For this reason, most of the current research in the field of nanocomposite materials focuses on thin films and coatings,^32,33^ for which only low quantities of NPs are required, but still proving their unique functionality.

The presence of NPs embedded in *e.g.* polymers, which are often chosen because of their wide variety of chemical and mechanical properties, raises new challenges for characterizing the composition and spatial homogeneity of thin films.^29,34^ Traditional characterization methods, which rely on mechanical and optical properties of composites, are not always applicable, as they occasionally provide only indirect information about the composition and cannot always determine the spatial distribution of NPs.^2^ Microscopy based techniques are powerful and can provide detailed information but are time consuming, at times destructive and require expensive tools, such as, *e.g.*, energy dispersive X-ray spectroscopy (EDX) and focused ion beam (FIB).^35,36^ Scattering techniques have also been used, but they are often hard to interpret and heavily rely on complex mathematical models to extract information about NPs.^37,38^ Methods based on elemental analysis are also employed to determine the composition of nanocomposites; they are accurate and reliable but only applicable to certain elements (typically metals), destructive, and expensive.^39,40^

Lock-in thermography (LIT) is an emerging non-destructive analytical technique for the investigation of stimuli-responsive nanomaterials.^41,42^ It is conventionally employed in the analysis of solid films, especially for applications in solar cells, and has the unique ability of providing a two-dimensional image of the sample emitting heat, thus allowing the assessment of the homogeneity of the samples. LIT makes use of the ability of some materials, such as plasmonic NPs or, in general, strongly optically absorbing components, to efficiently convert the absorbed energy of incident radiation into thermal energy.^41,43^ The detection will depend on various factors, including the thermal properties of the material, the concentration of the absorbing material and the sensitivity of the IR-camera. LIT allows the detection of temperature variations on the surface of the sample in the millikelvin range.^44^ Unlike conventional thermography, which simply records the temperature change of a sample upon illumination with monochromatic light, LIT works by modulating the intensity of the incident radiation at a given frequency and by recording the amplitude of the first harmonic of the temperature signal. By recoding it over numerous cycles, a high sensitivity can be achieved, while keeping the measurement time very short. This approach allows the signal-to-noise ratio to be significantly improved and the spurious effects of background noise to be eliminated.^41,42,44^ Other examples of thermographic techniques are vibrothermography (also known as ultrasonic thermography),^45^ pulsed thermography (PT)^46^ and pulsed phase thermography (PPT).^47^ The main difference from LIT is the excitation process. PT and PPT use high-energy lamps to uniformly heat the specimen surface for a finite time and then record the temperature decay over time by means of an infrared camera. A Fourier analysis of the signal is then applied to extract information about the sample. Contrary to traditional optical excitation thermography, vibrothermography employs mechanical waves to directly stimulate the sample. These techniques find application in the detection and visualization of subsurface flaws in large composite materials, such as carbon fibre reinforced polymers in industrial structures (*e.g.*, aircraft, vehicles, ships, and sports equipment). LIT represents a valuable alternative method to investigate NPs in complex systems, such as multicomponent suspensions, biological fluids and composite materials, with the great advantage of being a simple, non-destructive, and time- and cost-effective technique compared to microscopy and ellipsometry. Indeed, LIT measurements can be carried out within minutes and do not require expensive setups, experienced users or complex data analysis.^41^ The use of LIT has been recently demonstrated for the qualitative^48^ and quantitative^49^ determination of gold (Au) and silver (Ag) plasmonic NPs in suspensions. Moreover, LIT was successfully employed to detect and discriminate between aggregated and non-aggregated plasmonic NPs.^48^ Additionally, LIT has also been used to quantify the heat generated by superparamagnetic iron oxide nanoparticles (SPIONs) exposed to an alternating magnetic field.^50,51^

In this work, we aim at extending the application of LIT as an analytical method to detect and quantify gold nanoparticles (AuNPs) dispersed in a polymer film. The investigated system consists of polyvinyl alcohol (PVA) thin films, prepared by evaporation and containing different concentrations of AuNPs. By using LIT measurements, we were able to quantitatively measure the concentration of plasmonic NPs in the solid-state. This is accomplished by using a theoretical model that corroborates the experimental data and that can predict the heat generated for a known NP concentration. The model can be used for optimizing sample preparation and experiment design. Our results bring new advancements in the development of LIT as a new technique for the determination and characterization of NPs embedded in complex environments.

## Experimental section

### Synthesis of gold nanospheres (AuNPs@PVP)

The Turkevich method was used to synthesize gold nanoparticles (AuNPs).^52^ Namely, a suspension of 0.5 mM gold (99% tetrachloroauric acid, HAuCl_4_·3H_2_O, Sigma-Aldrich, Switzerland) and 1.5 mM of sodium citrate (≥98%, C_6_H_5_Na_3_O_7_·2H_2_O, Sigma-Aldrich, Switzerland) was brought to boiling and subsequently cooled down to room temperature resulting in the formation of 16 nm AuNPs capped with citrate. AuNPs of 41 nm were synthesized through the reduction of gold salt (0.25 mM) mixed with hydroxylamine hydrochloride (0.2 M) in the presence of sodium citrate (0.5 mM) and as-prepared citrate capped 16 nm AuNPs (0.0125 mM). The two AuNP size-types synthesized were surface-functionalized with polyvinylpyrrolidone (PVP, 8 kDa (C_6_H_9_NO)_*n*_, Acros Organics, Switzerland), by mixing 0.47 mM of Au sols with 2.65 mM PVP aqueous solution for the 16 nm AuNPs (labelled as Au16) and with 1.1 mM PVP for the 41 nm AuNPs (labelled as Au41).^53^

### Preparation of nanoparticle-embedded films

Polyvinyl alcohol (PVA 85–124 kDa, Sigma-Aldrich, Switzerland) films with embedded AuNPs were prepared as follows: 1 g of PVA powder was mixed with 9 mL of Milli-Q water and heated to 70 °C and stirred at 350 rpm overnight. Subsequently, the viscous liquid was cooled down for 5 min under continuous stirring and then 500 μL of a NP dispersion containing either Au16 or Au41 at a defined concentration were rapidly added. After an additional 5 min of cooling and stirring, the mixture was gently extracted by using a syringe and dropcast into a petri dish (*ϕ* 88 mm; 4 mL dropcast) or an aluminium sample pan (*ϕ* 90 mm; 7 mL dropcast). The PVA/Au films were then cured overnight at 50 °C.

### Lock-in thermography (LIT)

Experimental LIT measurements were carried out using a commercial LIT setup (Calorsito VIS-NIR, NanoLockin, Switzerland), including a homogeneous multi-wavelength LED-based light source (with the following available wavelengths: 400, 460, 525, 660, 730 and 940 nm), which is capable of exciting a broad variety of NP types depending on their specific absorbance spectra. LEDs with a wavelength of 525 nm (having a power density of 91 mW cm^−2^) were used for AuNPs; all samples were illuminated from below. Measurements were performed by using two custom-made sample holders designed for the films and liquid samples following ref. 54. For liquid samples, 80 μL of NP dispersion in a water dispersion were placed in between two IR transparent windows (barium fluoride windows) in a Teflon measurement cell sealed using 6 screws. Typically, a modulation frequency of 1 Hz and 180 seconds of measurement time are used. However, several modulation frequencies, ranging from 0.25 to 8 Hz have been used. The sample holder for the films consists of two magnetic rings in between which a single film was placed and kept flat to avoid artefacts. For statistical significance and to ensure reproducibility, each measurement was repeated five times. By placing a light-homogenizing glass rod (N-BK7, Edmond Optics, USA) directly between the LED panel and the sample holder, homogeneous illumination of the dispersions was ensured. Analysis of the resulting amplitude images was carried out to extract signal mean and standard deviation values (ImageJ v1.52b, NIH, USA & Origin 2016, OriginLab, USA).

### UV-vis spectroscopy (UV-vis): reflectance and transmittance measurements

Reflectance and transmittance spectra were acquired with an integrating sphere (AvaSphere-30, Avantes BV, Apeldoorn, Netherlands), connected with optical fibers to a flame UV-vis spectrophotometer (Ocean Optics, Dunedin, FL, USA) and a balanced deuterium-halogen lamp (DH-2000-Bal; Ocean Optics, Dunedin, FL, USA). Spectra were recorded after acquiring the dark and reference measurements.

### Transmission electron microscopy (TEM)

For the characterization of NP core diameter, a Tecnai Spirit transmission electron microscope (FEI, USA) operating at 120 kV was used. The samples were prepared by applying 10 μL of NP dispersion on a parafilm and subsequently placing 200 mesh Cu grids with continuous carbon films on top for 10 min and then drying overnight. TEM images were recorded with a Veleta CCD camera (Olympus, Japan), and evaluation of the images was performed with ImageJ (v1.52b, NIH, USA).

### Scanning electron microscopy (SEM)

A Tescan Mira 3 scanning electron microscope was used for SEM image acquisition. SEM image analysis was performed with ImageJ (v1.53u, NIH, USA). For the sample preparation, small pieces of films were cut with a scalpel and stuck to standard 90° pin stubs. The film thickness was determined by manually measuring the film cross-section of more than 15 areas per sample. The measured values are reported in Table 2. The average length for each film was taken as the film thickness, and ± indicates the standard deviation of the mean.

### Dynamic light scattering (DLS)

The hydrodynamic diameter of the prepared NPs in dispersion was measured by dynamic light scattering (DLS) using a 90 Plus particle size analyzer (Brookhaven Instruments, USA). The instrument operates with a 633 nm laser and measures the autocorrelation function at a scattering angle of 90°. The autocorrelation function is fitted using the cumulant method, from which the hydrodynamic diameter and the polydispersity index are obtained.

### Mathematical model

The modelling approach used here to quantify the presence of absorbing NPs inside a polymer film differs slightly from the one previously developed for the quantification of the heat generated by NPs in a liquid dispersion.^49^ It will be assumed that the thickness of the film is sufficiently small, so that temperature gradients along the film thickness can be neglected. Under the assumption of uniform temperature inside the film, if the NP distribution is also uniform inside the film and the intensity of the incident light is also spatially uniform, a simple and ordinary differential equation for the time evolution of the temperature inside the film can be written, arising from the energy balance:^49^1
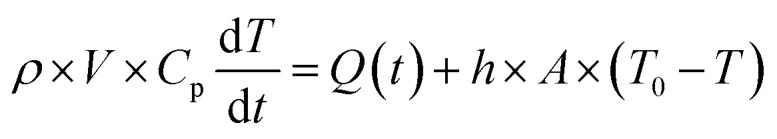


The solution of the equation can be obtained if the initial temperature *T* equals the outer temperature *T*_0_ at time *t* = *0*. In eqn (1), *h* is the heat transfer coefficient, *A* is the heat exchange area, *Q* is the power generated by the light absorbed by the film, *ρ* is the sample density, *V* the sample volume and *C*_p_ is the heat capacity of the sample at constant pressure. The equation can be written in dimensionless form as follows:2
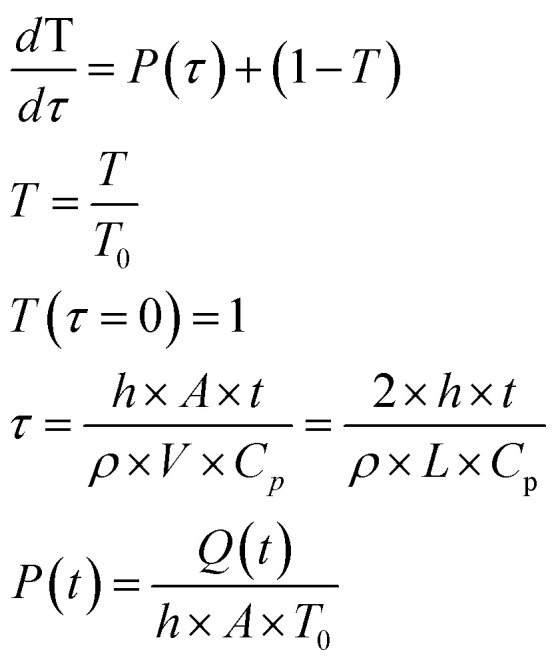
where *τ* is the dimensionless time and *P* is the dimensionless generated heat power. Note that in the fourth equation of eqn (2), *i.e.*, the one defining *τ*, the ratio between surface and volume has been set to be equal to twice the reverse thickness of the sample, because heat is dissipated by the film from both surfaces, the upper and the lower one. The heat balance differential equation is linear and can be solved exactly, as long as the time dependence of the generated power is known. One should consider that the intensity of the incident radiation is square modulated:3
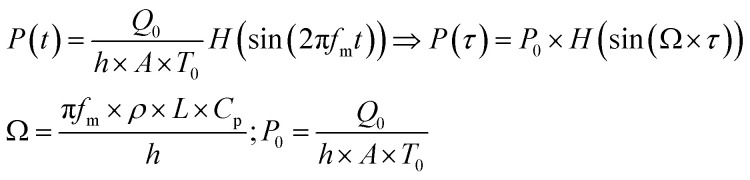
where *Q*_0_ is the heat power generated in the absence of modulation and *L* the thickness of the film, *Ω* is the dimensionless modulation frequency, *H*(*x*) is the Heaviside step function, and *P*_0_ the dimensionless generated power amplitude. The approach used to account for the heat dissipated by a solid thin film is obtained from optics and is applicable under the assumption that scattering caused by the NPs is much weaker than the absorption, which is certainly the case for the small NPs with strong plasmonic behavior used in this work. We will use Fresnel's formulas to compute the reflectivity and transmissivity of the film in the case of radiation with perpendicular incidence to the film surface.^55^ The fraction of the incident radiation converted to heat is proportional to the absorbance of the film and will be computed as the difference between the fraction of light transmitted through the film (*i.e.*, the film transmittance) and the fraction of light reflected by the sample (*i.e.*, the film reflectance). The reflectance *R* and transmittance *T*_r_ are expressed in terms of the reflective indices *n*_1_ = 1 of air, *n*_2_ of the film and *n*_3_ = *n*_1_ of air assuming that the film is free-standing and that it is in contact with air on both sides. The equations to obtain the transmittance and the reflectance for a film with thickness *L* and a radiation with wavelength *λ* are the following:^55^4
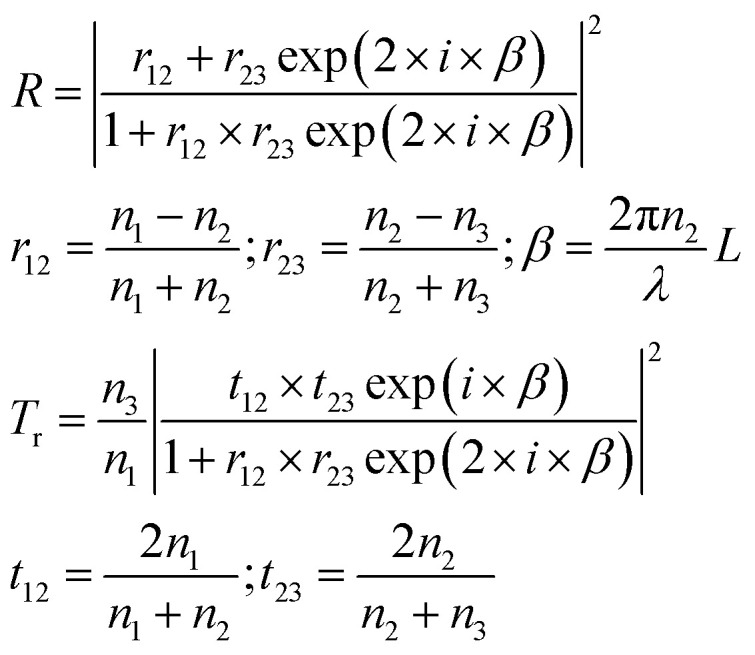


It should be remembered that the refractive index of the film *n*_2_ is generally a complex number whose real and imaginary parts depend on the wavelength. The overall incident light intensity is split into three contributions: a reflected part (*R*), a transmitted part (*T*_r_) and an absorbed part (*A*_b_). Therefore, the quantity of heat generated (*Q*_0_) by the film illuminated by the radiation is equal to the product of the intensity of the incident radiation (*I*_0_), the absorbance *A*_b_ of the film and the area. Therefore:5
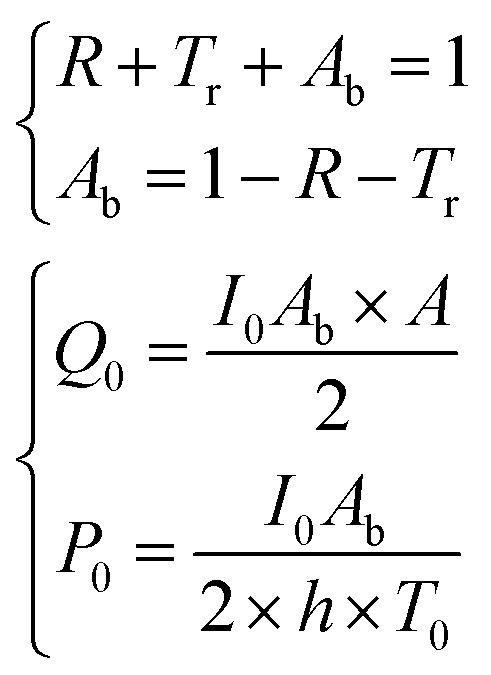


The factor two at the denominator is due to the fact that the surface over which the incident radiation impinges on the sample is half of the heat exchange surface *A*, because the heat is removed from both sides of the film, while the incident radiation illuminates only one side of the film. Since for the LED radiation used in our work the incident radiation is not monochromatic, the intensity spectrum has been measured as a function of the wavelength, and the overall dimensionless power amplitude must be integrated over the entire range of wavelengths *λ*:6
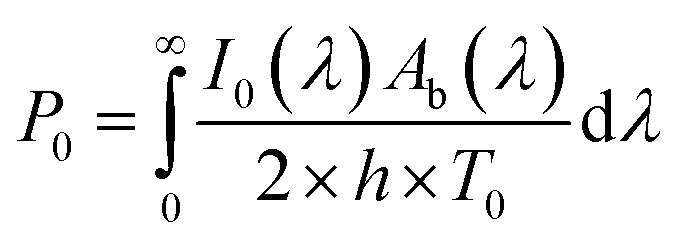
In the case of a homogeneous material, all quantities can be computed when the film material refractive index dependence on the wavelength is known. The situation is more complicated in the case of composite materials, because a model must be used to compute the refractive index of the film from its composition and structure. There are a variety of models available, starting from Maxwell–Garnett theory. When a composite material is filled with spherical nanoparticles with small size compared to the wavelength of the incident radiation, and when their concentration is not very high, as in the case of the materials characterized in this work, most of the models give similar results. For a review of various models, the publication by Stoyanov *et al.* is a good reference.^56^ In this work, we will use the effective field approximation theory. This model uses an expression for the dielectric constant of the composite material *ε*_c_, given the dielectric constants of the dispersed phase *ε*_p_ and of the medium *ε*_m_, the volume fraction of the dispersed phase *ϕ*, and the radius of the dispersed particles *R*_p_. From the dielectric constant of the composite medium, the refractive index is computed as its square root:^56^7
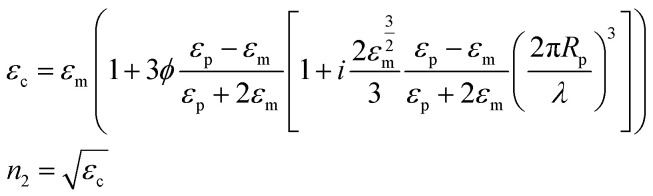
In eqn (7), *i* is the imaginary unit. More complex models have been tested,^56^ but no differences in the range of concentrations explored in this work have been found. The expression for the dielectric constant of gold as a function of wavelength was obtained from the literature.^57^ One should be aware that the overall balance used in eqn (5) is valid as long as the scattering contribution of the NPs embedded in the polymer matrix is negligible, which is the case when the size of the gold NPs is much smaller than the wavelength of the incident radiation. The real and imaginary parts of the reflective index of PVA were obtained by simultaneously fitting the measured transmittance and reflectance of a pure PVA film using the third equation of eqn (4) with the refractive index as a fitting parameter. The real part of the refractive index has then been compared with data from the literature^58^ and found to be in excellent agreement. Once the expression of the dimensionless power as computed from eqn (3) and (5) is known, eqn (2) can be solved, as performed in our previous publication.^49^ The final result for the amplitude of the first harmonics, measured in units of temperature, in the case of a square modulation, is the following:8
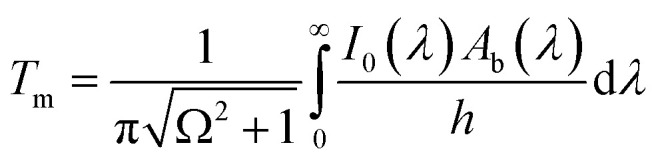


Under adiabaticity conditions, *i.e.*, when heat transfer is negligible (*h* → 0), eqn (8) reduces to:9
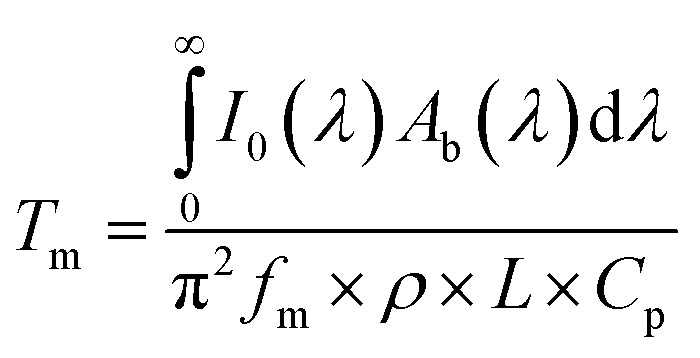


This equation indicates that the amplitude is inversely proportional to the modulation frequency. Therefore, by plotting the amplitude as a function of the reverse frequency, a linear trend is obtained, with a slope proportional to the generated heat.

## Results and discussion

The objective of this work is to demonstrate that LIT can be used to quantitatively account for the heat generation of NPs embedded in a polymer matrix. For this purpose, AuNPs with two different sizes have been prepared by a modification of the Turkevich method.^52^ The smallest NPs (referred to as Au16) have been prepared in a one-step synthesis, while the largest ones (referred to as Au41) have been prepared by using a seeded-growth method using Au16 as seeds (see Materials and methods for details). Both NPs possessed a moderately narrow size distribution and a predominantly spherical shape, as shown by TEM analysis (Fig. S1†). Image analysis performed over several TEM images and counting over 300 particles (Au16) and over 160 particles (Au41) led to an average Feret diameter of 15.9 nm and of 40.6 nm for the Au16 and the Au41 nanoparticles, respectively (Table 1). The size and aggregation state of the AuNPs in suspensions have been measured by DLS. The results of DLS measurements indicate a hydrodynamic diameter of about 23.9 nm and of 43.2 nm for the Au16 and the Au41 nanoparticles, respectively (Table 1). The fact that the hydrodynamic diameter of the particles is higher than the TEM diameter is due to both DLS being more sensitive to larger particles in the population than TEM, from which a number average diameter is obtained, and to the PVP coating on their surface, which is responsible for a size increase of a few nanometres. All data show that AuNPs are well dispersed in solution, possess a rather narrow size distribution and do not display any detectable aggregation. In a previous publication, we have shown that heat generated by NPs does not depend on their size if they are not aggregated, nor on their shape as long as it does not deviate significantly from a spherical shape, for particles smaller than 50 nm.^49^ Therefore, for the calculations of the generated heat, only the mean Feret diameter of the nanoparticles has been used.

**Table 1:** Characterization of AuNP size, determined from image analysis of TEM micrographs and from dynamic light scattering (DLS)

Nanoparticle designation	Smallest Feret diameter (nm)	Largest Feret diameter (nm)	Mean Feret diameter (nm)	Hydrodynamic diameter (DLS) (nm)	PDI (DLS)
Au16	14.5 ± 2.7	17.3 ± 3.6	15.9 ± 3.0	23.9	0.2
Au41	36.8 ± 8.2	44.4 ± 9.5	40.6 ± 8.6	43.2	0.2

**Fig. 1:**
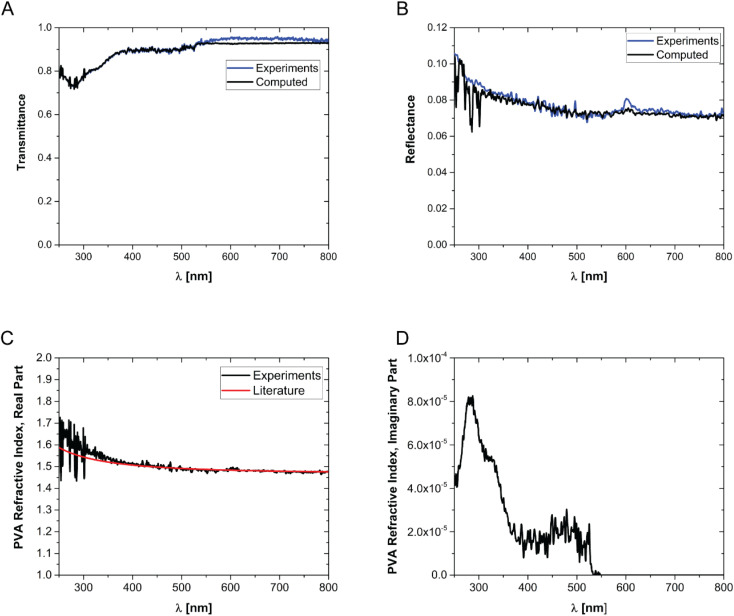
Transmittance (A) and reflectance (B) of pure PVA films. (C) Real part and (D) imaginary part of the refractive index of PVA. The data are obtained by fitting the reflectance and transmittance spectra of pure PVA films. The red line in (C) shows the real part of the PVA refractive index from the literature.^55^

**Fig. 2:**
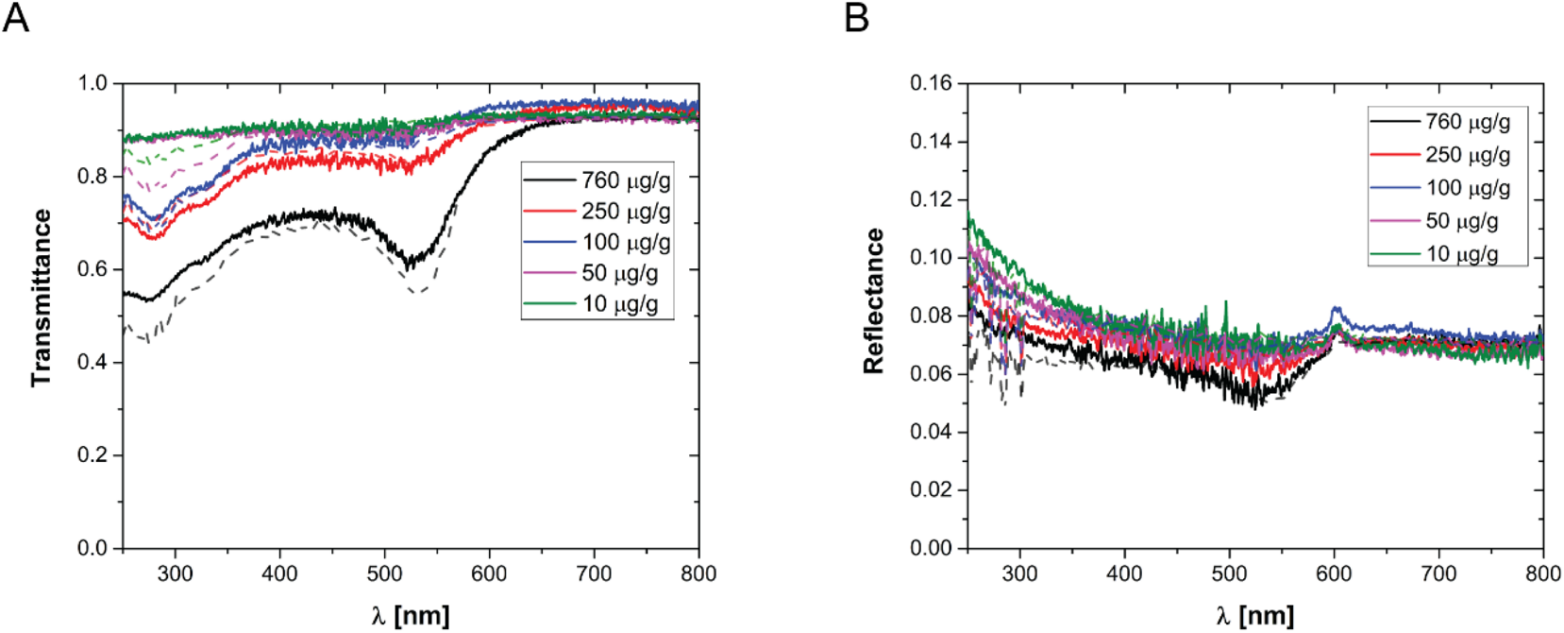
(A) Transmittance and (B) reflectance of various PVA/Au films, with different concentrations of AuNPs per gram of polymer, as indicated in the legend. The dots show the experimental data, while the lines show the corresponding model predictions from eqn (4).

In order to prepare films with NPs uniformly dispersed within the film, AuNPs at a desired concentration were dispersed in an aqueous solution containing a fixed mass fraction of PVA (10 wt%). Then, an aliquot of the suspension was dropcast in a suitable mould and the water was evaporated in an oven at a constant temperature of 50 °C. The evaporation temperature was sufficient to completely remove the water without forming bubbles in the films and to obtain uniform PVA/Au films. The thickness of the films has been measured by SEM, by imaging the cross-section of the films at different positions, and an average value has been obtained (for details see Fig. S2† and Table 2). The concentration of nanoparticles inside the film has been changed from 10 to 760 μg of gold per gram of polymer. In order to verify the absence of AuNP aggregates, the transmittance and reflectance of UV-vis spectra of the PVA/Au films have been measured using an integrated sphere UV-vis spectrometer (Fig. 2). As explained in Section 2.8, the transmittance and reflectance spectra of pure PVA were used to obtain both the real and the imaginary parts of the refractive index of PVA. The data are shown in Fig. 1C and D, together with literature values for the real part of the refractive index (red line in Fig. 1C). It is observed how the real part of the PVA refractive index extracted from the experimental data is almost identical to the literature data, thus confirming the reliability of our data and of the approach used. One can furthermore observe that the imaginary part of the refractive index of PVA is most important at low wavelengths, while in the region of the spectrum used for LIT measurements (around 525 nm) it does not have any significant contribution. The transmittance and reflectance spectra of the composite films are shown in Fig. 2A and B, respectively. One can notice that the plasmonic peak of the AuNPs does not shift from aqueous dispersions of the same NPs^49^ to the solid films and no additional peaks, which would indicate the presence of clusters, are noticeable in the films. Furthermore, one can also notice that, at the lowest concentration, the presence of the plasmonic peak of the particles is not visible. As we will show later on, LIT can clearly detect the heat released by the NPs even at such low concentrations, thus showing the sensitivity of the technique. In Fig. 2A and B the predictions of the optical model used to compute both transmittance and reflectance of PVA/Au films, according to eqn (4) are also shown. The good agreement of the model with the experimental data, without adjustable parameters, supports the reliability of our theoretical approach.

**Table 2:** Parameter values used in the calculations of LIT amplitude for the various samples. The PVA/Au film thicknesses are reported as mean ± standard deviation of the mean

Parameter	Value	Ref.	Au nanoparticles embedded
Heat transfer coefficient *h*	6 (Wm^−2^ K)	59	
PVA density *ρ*_p_	1190 (Kg m^−3)^	60	
PVA specific heat capacity *c*_p_	1675 (J Kg^−1^ K^−1^)	60	
PVA/Au 760 μg g^−1^ thin, thickness *L*	35.4 ± 2.5 (μm)	SEM image analysis	Au41
PVA/Au 760 μg g^−1^ thick, thickness *L*	82.5 ± 0.9 (μm)	SEM image analysis	Au16
PVA/Au 250 μg g^−1^, thickness *L*	51.4 ± 1.1 (μm)	SEM image analysis	Au16
PVA/Au 100 μg g^−1^, thickness *L*	65.3 ± 1.5 (μm)	SEM image analysis	Au16
PVA/Au 50 μg g^−1^, thickness *L*	40.1 (μm)	SEM image analysis	Au41
PVA/Au 10 μg g^−1^ thickness *L*	21.9 ± 3.1 (μm)	SEM image analysis	Au41
PVA thickness *L*	55.7 (μm)	SEM image analysis	—

**Fig. 3:**
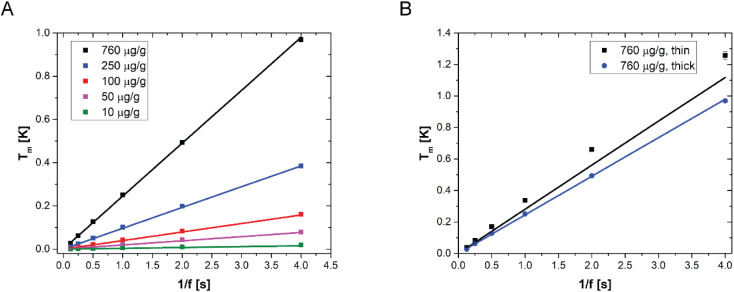
(A) LIT measured temperature amplitude (squares) as a function of the reverse modulation frequency, for various films with different AuNP concentrations, sizes and thicknesses, as reported in Table 2. (B) LIT measured temperature amplitude for two different samples with an identical nanoparticle concentration (760 μg g^−1^) but different thicknesses. In both figures, the lines are the model predictions according to eqn (8).

LIT measurements have been carried out at various modulation frequencies (see Fig. S3† for representative LIT images). The results of the experiments are shown in Fig. 3A together with the model predictions obtained from eqn (8). The values of the various physical quantities used in the model are reported in Table 2, with the corresponding references. One can observe that the model predictions are in good agreement with the experimental data for all NP concentrations and for all the modulation frequencies. Furthermore, it appears from the linearity of the temperature amplitude as a function of the reverse modulation frequency shown in Fig. 3 that the measurements performed can be simulated under the assumption of adiabaticity of the system (which means that the heat transfer coefficient would be equal to zero). The very low heat transfer coefficient value used to simulate the data, which is consistent with heat transfer by natural convection, confirms this observation. It should be noted that some of the films have been prepared with Au41 NPs, while others were prepared with Au16 NPs (see Table 2 for details). One can observe that the model predicts that the LIT signal is independent of the particle size, consistent with what has already been observed with UV-vis transmittance and reflectance spectra. Furthermore, Fig. 3B shows two sets of experimental data, corresponding to films prepared with the same gold concentration (the highest one used in this work), but with different NP sizes and thicknesses. Interestingly, the model predicts quantitatively the different heat generated by the two samples, which is due to the sample thickness.

**Fig. 4:**
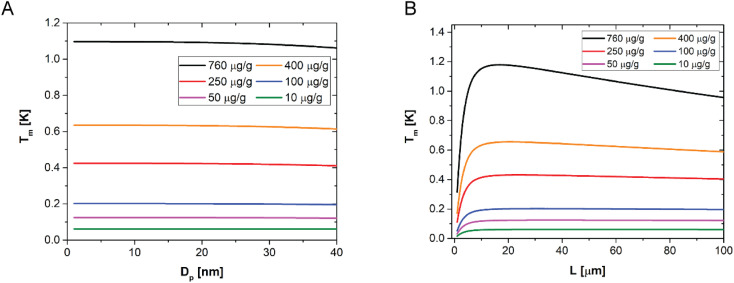
(A) Computed temperature amplitude (*T*_m_) as a function of the NP diameter *D*_p_, for different concentrations of NPs as indicated in the legend, for a fixed film thickness, equal to 50 μm, and for a fixed modulation frequency of 0.25 Hz. (B) Computed temperature amplitude (*T*_m_) as a function of the sample thickness, for different concentrations of NPs as indicated in the legend, for a fixed NP size, equal to 15 nm, and for a fixed modulation frequency of 0.25 Hz.

A sensitivity analysis of the model over the NP size up to 40 nm is shown in Fig. 4A. It confirms that, for a given concentration and sample thickness, the temperature amplitude does not depend on the particle size, for all the concentrations investigated in this work. This happens because the AuNP concentration is sufficiently low to neglect interparticle interactions in the composite films as well as nanoparticle scattering contributions. Fig. 4B, instead, shows the sensitivity of the model to the sample thickness, for a given NP size (1–40 nm) and for different NP concentrations, at a given modulation frequency. One can observe that there is a small dependence on the sample thickness only measurable in the case of the highest concentration of particles used in this work, and even in this case the maximum difference in amplitude is about 20%. For all the lower concentrations, no dependence is observed. These results suggest that determining the thickness of the sample from LIT measurements is in general not possible, while a very accurate determination of the concentration of particles is possible. The reason behind the low sensitivity of the LIT measurements on the sample thickness is due to a combination of the dependence of the dissipated heat on the thickness, which can be assumed to be proportional to the absorbance of the light going through the material. The absorbance is proportional to:^55^

10 *A*_b_ ∼1 − exp(−*α* × *L*) ∼ *α* × *L*

where the absorption coefficient *α* depends on the concentration of gold inside the material. When the term in the exponential is not too large, a Taylor expansion can be used as shown in eqn (10). This means that as long as the Taylor expansion can be truncated at the first order term, the absorbance is linearly proportional to the thickness and that the temperature amplitude measured by LIT is independent of the thickness, as the term proportional to the sample thickness *L* in eqn (10) is compensated for by the corresponding term in the denominator of eqn (8). As the concentration of gold increases, *α* increases as well, leading to higher order terms becoming relevant, which leads to a decrease in the LIT signal with the sample thickness. When instead the sample thickness decreases to very small values, the condition of adiabaticity is not verified anymore, because the surface to volume ratio of the sample increases enough for heat transfer to become relevant even with very low heat transfer coefficients. This explains the first part of the curves in Fig. 4B, showing an increase in the amplitude as a function of the sample thickness. Therefore, according to these results and our model predictions, a determination of the sample thickness from LIT measurements is only possible for very thin samples, as Fig. 4B indicates.

**Fig. 5:**
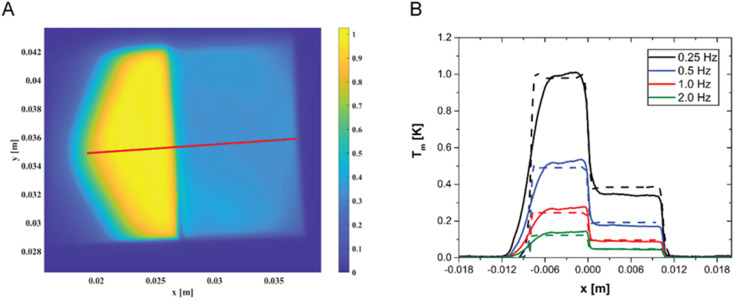
(A) Two-dimensional heat map measured by LIT of a sample with a concentration step, showing the strong difference in the signal between the two parts, with different concentrations (760 μg g^−1^ and 250 μg g^−1^, respectively) of gold nanoparticles. The numbers on the *x* and *y* axes are the lengths in meters. (B) Experimental (dashed lines) and simulated (continuous lines) LIT-measured profiles along the red line shown in (A) of a sample with a gold nanoparticle concentration gradient: 760 μg g^−1^ for *x* < 0 and 250 μg g^−1^ for *x* > 0.

In order to further prove the reliability of LIT as an analytical tool and to further validate the model, we have prepared a sample with a concentration step, obtained by gluing together two samples: 760 μg of AuNPs per gram of polymer on the left and the 250 μg AuNPs per gram of polymer on the right. A typical LIT image of such a sample is shown in Fig. 5A. LIT is very sensitive to concentrations, and the image clearly shows the presence of a sharp step in the recorded signal at the junction between the two samples. The portion of hexagonal shape shown on the left-hand side of the sample is due to the shape of the LED panel used to illuminate the sample having a hexagonal cross-section. It is easily possible to create a model that predicts the presence of such a concentration step by extending the modelling to account for the spatial dependence of the concentration. The predictions of the model are shown in Fig. 5B in comparison with the measured data, taken along the red line shown in Fig. 5A, and no adjustable parameters have been used in the simulations. The agreement is very good for all modulation frequencies, with the exception of the region on the left-hand side of the figure, which corresponds to the region where the LED panel reaches its boundary (and which is difficult to model exactly, because the light intensity decays close to the LED panel border, and such decay has not been measured). The highly satisfactory agreement between experimentally measured LIT temperature amplitude and simulations clearly shows the reliability of the developed approach. Moreover, this approach suggests that it would also be possible to detect and quantify concentration gradients.

## Conclusions

In this work, we have shown how lock-in thermography can be used to detect and quantify AuNPs embedded in thin polymer films. Composite films were prepared from aqueous suspensions of PVA and AuNPs (average particle sizes of about 16 and 41 nm), dropcast in a suitable mould, from which water was slowly evaporated to ensure uniform dispersion of the particles inside the polymer. The AuNPs have been thoroughly characterized by TEM and DLS to determine their size, size distribution and the possible presence of aggregates. UV-vis spectroscopy was also used to measure the transmission and reflection of the samples. Pure PVA films were used to determine the real and imaginary parts of the polymer refractive index, for which only data on the real part are available in the literature. The film thicknesses were characterized using SEM. Subsequently, the films were exposed to modulated light intensity with a wavelength close to that of the plasmonic peak of the nanoparticles, and their thermal signature was measured for different modulation frequencies. The method was shown to be capable of detecting the presence of very small concentrations of NPs embedded in the polymer matrix, which is not possible with UV-vis spectroscopy. A quantitative mathematical model has been developed, based on Fresnel formulas for calculating the absorption of nanocomposite films. This model has been proven to quantitatively predict the amplitude of the LIT signal, for all particle concentrations investigated in this work. Sensitivity analysis has shown that the amplitude signal from LIT is insensitive to sample thickness, as long as the thickness is more than 10 μm and the concentration does not exceed 400 μg g^−1^ of polymer. Above this threshold, an increase in thickness leads to a measurable decrease in LIT amplitude, as shown by the data reported in this work. In addition, the LIT amplitude has also been shown to be insensitive to the size of the AuNPs as long as they do not grow above 40 nm. Finally, a sample with an AuNP concentration gradient was prepared to show how strongly LIT responds to the presence of spatial concentration gradients and that the model is able to quantitatively describe the thermal signature of the concentration gradient. Therefore, the model presented here can be used as a valuable tool to predict the heat evolution at a known NP concentration, leading to better optimization of sample preparation and experiment design.

This work demonstrates that LIT is a powerful characterization technique that can non-destructively quantify the presence of NPs in solid thin films, even at very low concentrations, as long as they absorb radiation that can be converted into heat.

## Author contributions

A. P-F. and M. L. conceived the project. L. S. synthesised the nanoparticles and G. M. prepared all the films. G. M. performed the experiments. G. M. and M. L. analysed the data. M. L. developed the mathematical model and wrote the original draft of the manuscript. C. G., A. P-F., M. L. and M. B. were responsible for the acquisition of the financial support for the project. M. L., A. P-F., C. G., and M. B. supervised the project. All authors have contributed to writing – review & editing the manuscript. All authors approved the final version of the manuscript.

## Conflicts of interest

C. G. and M. B. have equity in the company NanoLockin GmbH, which specializes in lock-in thermal imaging instruments for NPs and various material analysis and might benefit from potential interest in this work.

## Supplementary Material

NA-005-D3NA00091E-s001

## References

[cit1] Chawla K. K. , Composite Materials: Science and Engineering, Springer, 4th edn, 2019

[cit2] Koo J. , Polymer Nanocomposites: Processing, Characterization, and Applications, McGraw Hill, 2nd edn, 2019

[cit3] Mahmoudi M., Sant S., Wang B., Laurent S., Sen T. (2011). Adv. Drug Delivery Rev..

[cit4] Lee K. S., El-Sayed M. A. (2006). J. Phys. Chem. B.

[cit5] Stewart M. E., Anderton C. R., Thompson L. B., Maria J., Gray S. K., Rogers J. A., Nuzzo R. G. (2008). Chem. Rev..

[cit6] Jain P. K., Huang X. H., El-Sayed I. H., El-Sayed M. A. (2008). Acc. Chem. Res..

[cit7] Linic S., Christopher P., Ingram D. B. (2011). Nat. Mater..

[cit8] Michalet X., Pinaud F. F., Bentolila L. A., Tsay J. M., Doose S., Li J. J., Sundaresan G., Wu A. M., Gambhir S. S., Weiss S. (2005). Science.

[cit9] Alivisatos A. P. (1996). Science.

[cit10] De Volder M. F. L., Tawfick S. H., Baughman R. H., Hart A. J. (2013). Science.

[cit11] Stankovich S., Dikin D. A., Dommett G. H. B., Kohlhaas K. M., Zimney E. J., Stach E. A., Piner R. D., Nguyen S. T., Ruoff R. S. (2006). Nature.

[cit12] Geim A. K., Novoselov K. S. (2007). Nat. Mater..

[cit13] Thostenson E. T., Ren Z. F., Chou T. W. (2001). Compos. Sci. Technol..

[cit14] Anker J. N., Hall W. P., Lyandres O., Shah N. C., Zhao J., Van Duyne R. P. (2008). Nat. Mater..

[cit15] Moon R. J., Martini A., Nairn J., Simonsen J., Youngblood J. (2011). Chem. Soc. Rev..

[cit16] Rycenga M., Cobley C. M., Zeng J., Li W. Y., Moran C. H., Zhang Q., Qin D., Xia Y. N. (2011). Chem. Rev..

[cit17] Alexandre M., Dubois P. (2000). Mater. Sci. Eng., R.

[cit18] Wang Z. L. (2004). J. Phys.: Condens. Matter.

[cit19] Wolf S. A., Awschalom D. D., Buhrman R. A., Daughton J. M., von Molnar S., Roukes M. L., Chtchelkanova A. Y., Treger D. M. (2001). Science.

[cit20] Lieber C. M., Wang Z. L. (2007). MRS Bull..

[cit21] Decher G. (1997). Science.

[cit22] Feng L., Li S. H., Li Y. S., Li H. J., Zhang L. J., Zhai J., Song Y. L., Liu B. Q., Jiang L., Zhu D. B. (2002). Adv. Mater..

[cit23] Lee H., Dellatore S. M., Miller W. M., Messersmith P. B. (2007). Science.

[cit24] Amjadi M., Kyung K. U., Park I., Sitti M. (2016). Adv. Funct. Mater..

[cit25] Someya T., Sekitani T., Iba S., Kato Y., Kawaguchi H., Sakurai T. (2004). Proc. Natl. Acad. Sci. U. S. A..

[cit26] Mannsfeld S. C. B., Tee B. C. K., Stoltenberg R. M., Chen C., Barman S., Muir B. V. O., Sokolov A. N., Reese C., Bao Z. N. (2010). Nat. Mater..

[cit27] Zhang J. J., Zou Q., Tian H. (2013). Adv. Mater..

[cit28] Chhowalla M., Shin H. S., Eda G., Li L. J., Loh K. P., Zhang H. (2013). Nat. Chem..

[cit29] Gangopadhyay R., De A. (2000). Chem. Mater..

[cit30] Brinker C. J., Lu Y. F., Sellinger A., Fan H. Y. (1999). Adv. Mater..

[cit31] Guozhong Cao Y. W. , Nanostructures and Nanomaterials: Synthesis, Properties, and Applications, World Scientific Publishing Company, 2nd edn, 2010

[cit32] Bruzzone A. A. G., Costa H. L., Lonardo P. M., Lucca D. A. (2008). CIRP Ann. Manuf. Technol..

[cit33] Wang Q. H., Kalantar-Zadeh K., Kis A., Coleman J. N., Strano M. S. (2012). Nat. Nanotechnol..

[cit34] Beecroft L. L., Ober C. K. (1997). Chem. Mater..

[cit35] David C. B. C. and Williams B., Transmission Electron Microscopy: A Textbook for Materials Science, Springer, 2009

[cit36] Joseph D. E. N. , Goldstein I., Michael J. R., Ritchie N. W. M., Scott J. H. J. and Joy D. C., Scanning Electron Microscopy and X-Ray Microanalysis, Springer, 2017

[cit37] Narayanan T., Konovalov O. (2020). Materials.

[cit38] Ding S. Y., Yi J., Li J. F., Ren B., Wu D. Y., Panneerselvam R., Tian Z. Q. (2016). Nat. Rev. Mater..

[cit39] Hahn D. W., Omenetto N. (2012). Appl. Spectrosc..

[cit40] Niehus H., Heiland W., Taglauer E. (1993). Surf. Sci. Rep..

[cit41] Bonmarin M., Steinmetz L., Spano F., Geers C. (2021). IEEE Instrum. Meas. Mag..

[cit42] Steinmetz L., Kirsch C., Geers C., Petri-Fink A., Bonmarin M. (2020). Nanomaterials.

[cit43] Baffou G., Quidant R. (2014). Chem. Soc. Rev..

[cit44] Breitenstein O. , Warta W. and Schubert M. C., Lock-in Thermography, Springer, Cham, 2018

[cit45] Liu B., Zhang H., Fernandes H., Maldague X. (2016). Sensors.

[cit46] Panella F. W., Pirinu A. (2021). J. Nondestr. Eval..

[cit47] Montinaro N., Fustaino M., Pantano A. (2020). Materials.

[cit48] Steinmetz L., Taladriz-Blanco P., Geers C., Spuch-Calvar M., Bonmarin M., Balog S., Rothen-Rutishauser B., Petri-Fink A. (2019). Part. Part. Syst. Charact..

[cit49] Steinmetz L., Geers C., Bonmarin M., Rothen-Rutishauser B., Petri-Fink A., Lattuada M. (2021). J. Phys. Chem. C.

[cit50] Monnier C. A., Crippa F., Geers C., Knapp E., Rothen-Rutishauser B., Bonmarin M., Lattuada M., Petri-Fink A. (2017). J. Phys. Chem. C.

[cit51] Lemal P., Geers C., Monnier C. A., Crippa F., Daum L., Urban D. A., Rothen-Rutishauser B., Bonmarin M., Petri-Fink A., Moore T. L. (2017). J. Magn. Magn. Mater..

[cit52] Turkevich J., Stevenson P. C., Hillier J. (1951). Discuss. Faraday Soc..

[cit53] Brown K. R., Natan M. J. (1998). Langmuir.

[cit54] Bredenbeck J., Hamm P. (2003). Rev. Sci. Instrum..

[cit55] Max Born E. W. , Principles of Optics: 60th Anniversary Edition, Cambridge University Press, 2020

[cit56] Stoyanov A. J., Howell B. F., Fischer E. C., Uberall H., Chouffani K. (1999). J. Appl. Phys..

[cit57] Trügler A. , Optical Properties of Metallic Nanoparticles, Springer, 2016

[cit58] Yovcheva T., Vlaeva I., Bodurov I., Dragostinova V., Sainov S. (2012). Appl. Opt..

[cit59] Byron Bird W. E. S. R. and Lightfoot E. N., Transport Phenomena, Revised, John Wiley & Sons, Inc., 2nd edn, 2006

[cit60] Thomas D., Zhuravlev E., Wurm A., Schick C., Cebe P. (2018). Polymer.

