# Supplementary material for: Quantification of nanoparticles' concentration inside polymer films using lock-in thermography
Source: Nanoscale Adv. 2023 Apr 27;5(11):2963–72. doi: 10.1039/d3na00091e (PMC10228360; doi:10.1039/d3na00091e)
Supplement: NA-005-D3NA00091E-s001 [file NA-005-D3NA00091E-s001.pdf]

Electronic Supplementary Material (ESI) for Nanoscale Advances.
This journal is © The Royal Society of Chemistry 2023

Supplementary Information

Quantification of nanoparticles' concentration inside polymer films using Lock-in Thermography

Giulia Mirabello, Lukas Steinmetz, Christoph Geers, Barbara Rothen-Ruthishauser, Mathias Bonmarin, Alke Fink and Marco Lattuada*

* Email: marco.lattuada@unifr.ch

Supplementary Figures

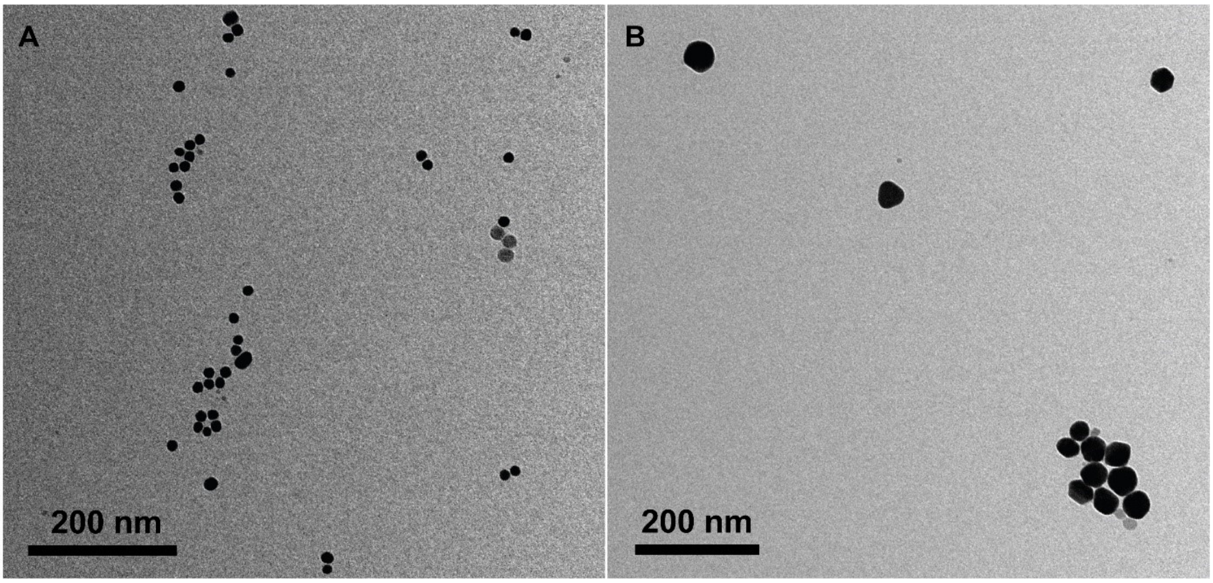

Fig. S1. TEM images of Au16 (left) and Au41 (right) nanoparticles, showing the quite narrow size distribution of the gold nanoparticles, and their regular shape, assumed to be spherical.

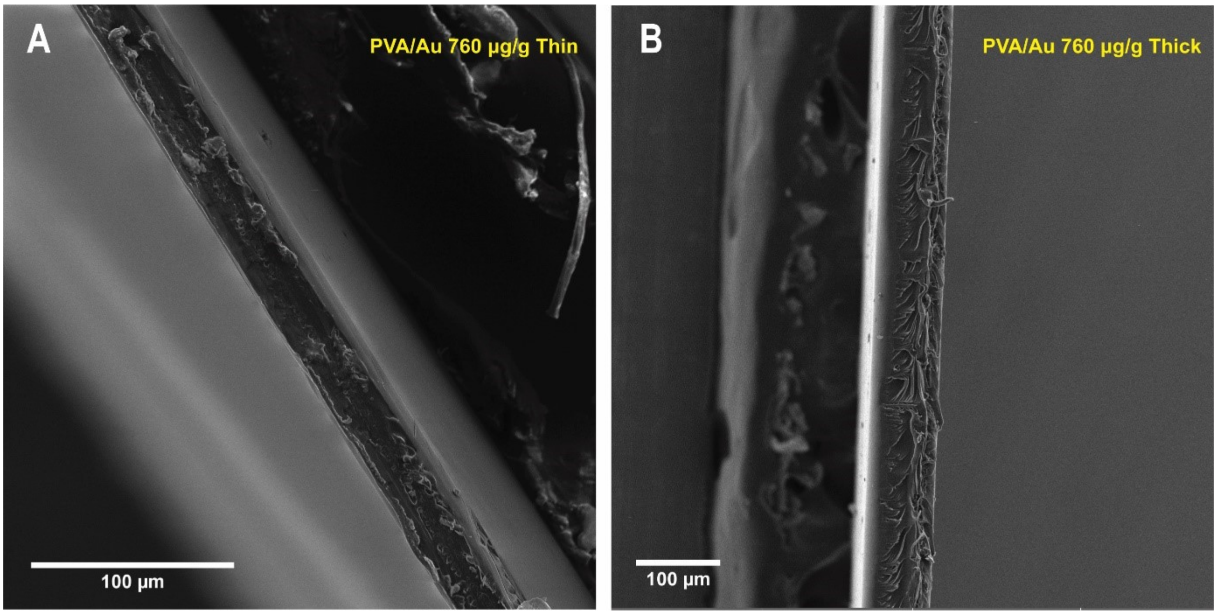

Fig. S2. SEM images of PVA/Au films. A) Representative SEM image of PVA/Au thin film containing 760 µg of Au41 NPs per gram of polymer. B) Representative SEM image of PVA/Au thick film containing 760 µg of Au16 NPs per gram of polymer.

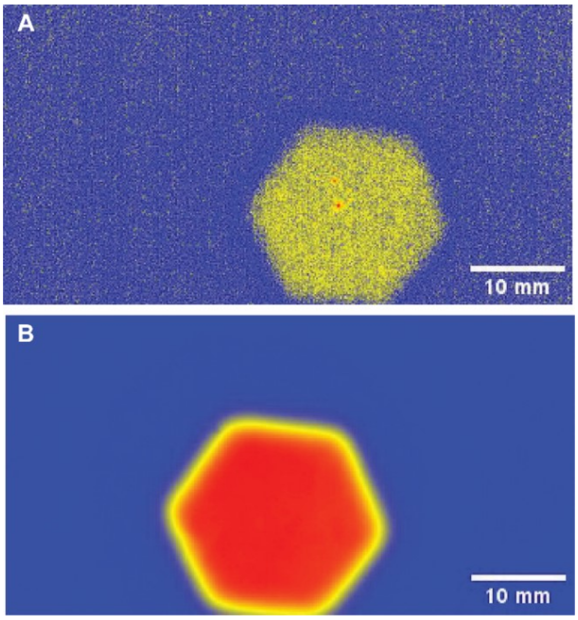

Fig. S3. Representative 2-D image of the temperature amplitude map of PVA/Au films acquired by lock-in thermography (LIT) at the modulation frequency of 1 Hz. A) LIT image of PVA/Au film containing 10 µg of Au16 NPs per gram of polymer. B) LIT image of PVA/Au thick film containing 760 µg of Au16 NPs per gram of polymer. The difference in temperature amplitude signal between A) and B) is due to the difference in AuNPs concentration.
